# Adaptability Promotes Student Engagement Under COVID-19: The Multiple Mediating Effects of Academic Emotion

**DOI:** 10.3389/fpsyg.2020.633265

**Published:** 2021-01-06

**Authors:** Keshun Zhang, Shizhen Wu, Yanling Xu, Wanjun Cao, Thomas Goetz, Elizabeth J. Parks-Stamm

**Affiliations:** ^1^Department of Psychology, Qingdao Psychological and Mental Health Research Institute, Normal College, Qingdao University, Qingdao, China; ^2^Student Counselling and Mental Health Center, Qingdao University, Qingdao, China; ^3^Department of Psychology, Normal College, Qingdao University, Qingdao, China; ^4^Department of Developmental and Educational Psychology, Faculty of Psychology, University of Vienna, Vienna, Austria; ^5^Department of Psychology, University of Southern Maine, Portland, ME, United States

**Keywords:** COVID-19, adaptability, student engagement, academic emotion, university student

## Abstract

In response to the COVID-19 pandemic, millions of students in China followed an emergency policy called “Suspending Classes without Stopping Learning” to continue their study online as schools across the country were closed. The present study examines how students adapted to learning online in these unprecedented circumstances. We aimed to explore the relationship between adaptability, academic emotion, and student engagement during COVID-19. 1,119 university students from 20 provinces participated in this longitudinal study (2 time points with a 2-week interval). The results showed that adaptability (the ability to respond to changes) and student engagement are significantly positively correlated with positive academic emotion and negatively correlated with negative academic emotion. Furthermore, adaptability not only directly predicts student engagement, but also affects student engagement through the chain mediation of positive academic emotion and negative academic emotion. The results contribute to the gap in knowledge regarding changes in students’ learning in response to the outbreak. This study further explains the internal mechanisms mediating the relationship between adaptability and student engagement. It may provide references for educational researchers and universities in dampening the negative effects of COVID-19 on students’ learning by improving their adaptability and developing positive academic emotions.

## Introduction

Beginning in late 2019, a novel coronavirus disease (COVID-19) spread widely and quickly around China and the world (Guan et al., 2020; Wu and McGoogan, 2020; Zhou et al., 2020). The pandemic brought not only the risk of death and illness, but also heightened stress, anxiety, and depression among individuals in China (Wang and Zhao, 2020; Wang et al., 2020). To contain the virus, the Ministry of Education of the People’s Republic of China initiated a series of emergency management steps, including shutting down schools and initiating online learning (i.e., Suspending Classes without Stopping Learning^1^). As a result, approximately 30 million quarantined university students experienced an unprecedented and unplanned switch from traditional face-to-face learning to online learning from home. The combined psychological pressure caused by COVID-19 and the abrupt change in learning modality created significant challenges for students, with significant impacts on the mental health of university students (Cao et al., 2020). However, no detailed study has examined the impact of university students’ adaptability on their learning during the COVID-19 pandemic. Thus, we conducted this study to investigate how adaptability (i.e., the ability to respond to changing, new, and uncertain conditions appropriately) may influence their engagement following the transition to online learning in China during the COVID-19 pandemic.

Student engagement refers to students’ active involvement in their learning and academic activities at school. Student engagement can be conceptualized as a three-dimensional construct, including behavioral, affective, and cognitive elements, which forms the basis of students’ connectedness to learning (Fredricks and McColskey, 2012). Students’ level of student engagement is associated with higher academic achievement (Appleton et al., 2008; Fredricks and McColskey, 2012) and mental health (Steele and Fullagar, 2009). Student engagement has been found to better predict the quality of higher education than resource input and the reputation of academies (Boulton et al., 2019).

As universities moved online in response to COVID-19, student engagement has been identified as a challenge (Farooq et al., 2020; Nickerson and Shea, 2020; Perets et al., 2020). However, very little research has examined the factors that influence student engagement in a pandemic. The present research examines the role of adaptability.

Adaptability is defined as the capacity to modify one’s cognition, affect, and behavior constructively, reflecting an individual difference in the way that one responds to changing, new, and uncertain conditions (VandenBos, 2007; Martin et al., 2012). There are three components: cognitive adjustment refers to the modification of one’s thoughts, behavioral adjustment refers to the modification of one’s actions, and affective adjustment refers to altering one’s affective responses (Martin et al., 2012; Holliman, 2013; Holliman et al., 2018). Recent research conducted during the COVID-19 pandemic found adaptability was predicted by students’ personality traits (Besser et al., 2020). Specifically, this research found that all five of the big five personality traits significantly predicted adaptability to the COVID-19 pandemic, with extraversion, openness, agreeableness, and conscientiousness positively associated with adaptability, and neuroticism negatively associated. There is also evidence that adaptability is related to student engagement. Martin and colleagues found that a higher level of adaptability was significantly associated with both greater positive student engagement and lower negative student engagement among secondary school students (Martin et al., 2013). Prior research has also found that 1st-year undergraduates’ adaptability was a significant direct predictor of both positive and negative behavioral engagement (Collie et al., 2016; Holliman et al., 2018). However, although previous research has examined the role of adaptability in transitions to new school environments (e.g., freshman in college or high school), no research has investigated the role of adaptability in students’ response to changes caused by a global pandemic. The evidence mentioned above suggests that adaptability may be an essential antecedent to students’ engagement in altered school environments during COVID-19.

The broaden-and-build theory of positive emotions suggests that the form and function of positive academic emotion and negative academic emotion are distinct and complementary (Fredrickson, 2001). Positive academic emotions can broaden the scope of individuals’ cognition and activities, and help students envision goals, challenges, and positive thoughts (Fredrickson, 2001; Fredrickson and Joiner, 2018). Students who search out learning opportunities and resources tend to have positive academic emotion. What is more, positive academic emotion can promote students’ persistence and efforts in learning (Pekrun et al., 2002). Therefore, positive academic emotions can make students feel more engaged in learning activities (Reschly et al., 2008). On the other hand, students with negative academic emotions (e.g., those who feel nervous, sad, anxious, bored, and disappointed) pay more attention to the threats in their environment, which limits their flexibility to invoke cognitive resources to perform at their best in learning activities (Derakshan et al., 2009). Therefore, negative academic emotions could exert a negative influence on students’ academic performance, which could further obstruct student engagement (Putwain et al., 2013). In sum, academic emotions appear to play an essential role in student engagement; whereas positive academic emotions tend to propel student engagement, negative academic emotions can impede student engagement (Zhen et al., 2017).

Furthermore, previous research has indicated that adaptability and academic emotions may work together to impact student engagement. When an individual is faced with novelty and uncertainty, he or she tends to make changes (e.g., in behavior, emotion, and cognition) to adapt to the new conditions. Whereas previous research in this field of adaptability have focused on changes in behavior and cognition (Collie and Martin, 2017), we examine the mediating role of emotion. Although some research has examined emotion regulation during the COVID-19 pandemic (Chen et al., 2020; Jiang et al., 2020; Restubog et al., 2020), the mediating role of emotions for the relationship between adaptation and school engagement is an important piece of the puzzle for understanding students’ response to the COVID-19 pandemic. Students who easily adapt to novelty and uncertainty experience positive academic emotions such as enjoyment and pride (Goetz et al., 2008), whereas students who feel unable to adapt are more likely to experience negative academic emotions, such as anxiety and boredom (King and Gaerlan, 2014).

In sum, this investigation has two aims: The first aim is to explore relations between students’ adaptability and engagement under COVID-19. The second aim relates to the function of academic emotions. Using a longitudinal design, we examine if adaptability influences academic emotions, and if academic emotions then influence student engagement in response to the wide-ranging changes in students’ academic experience during the COVID-19 pandemic, i.e., the possible mediating role of academic emotions.

## Materials and Methods

### Ethics Statement

This study was conducted based on the ethical standards in the WMA Declaration of Helsinki. The Research Ethics Committee of Qingdao University has approved this study. Participants were informed about the precise contents of the study before they started their assessments (including the goal of the research, duration, and anonymity in the surveys and data analyses). Furthermore, all identifiers that could link individual participants to their results were excluded in the data analyses; thus, all analyses were based on anonymous data.

### Participants and Design

We collected data via a Chinese online research panel, Wenjuanxing^2^, which provides functions equivalent to Amazon Mechanical Turk. Based on random sampling, 1,616 university students voluntarily participated in the Time 1 assessment and 1,119 of these participants took part in the Time 2 assessment (903 females, 216 male), with a 30.75% attrition rate. The age of the sample ranged from 17 to 37 years (*M* = 20.42, *SD* = 2.13). The final sample included 984 undergraduates and 135 postgraduates.

The study involved two assessments in the early stages of online learning. Time 1 (T1) took place from 29 February to 6 March 2020. Time 2 (T2) took place from 13 March to 20 March 2020. Both T1 and T2 focused on students’ adaptability, academic emotions, and academic engagement. Identical instruments were used at both times.

### Measures

#### Adaptability

We used the nine-item Adaptability Scale to assess students’ adaptability (Martin et al., 2012). This scale contains three items referring to affective adaptability (e.g., “When uncertainty arises, I am able to minimize frustration or irritability so that I can deal with it best”) and six items referring to cognitive-behavioral adaptability (e.g., “I am able to think through a number of possible options to assist me in a new situation”). The items were rated on a 7-point scale ranging from 1 (Strongly disagree) to 7 (Strongly agree). The scale was internally consistent (α_t__1__/t__2_ = 0.97/0.97).

#### Academic Emotion

We adapted subscales of the Differential Emotion Scale (Izard et al., 1974) to assess anger, sadness, fear, and enjoyment, and subscales of the Academic Emotions Questionnaire (Pekrun et al., 2002) to assess anxiety, hopelessness, and boredom. Each emotion includes three items, and thus 21 items in total were involved. Six negative differential emotions (i.e., anger, hopelessness, boredom, sadness, fear, anxiety; α_t__1__/t__2_ = 0.96/0.97) were grouped into one scale to measure negative academic emotions, and enjoyment (α_t__1__/t__2_ = 0.88/0.90) represented positive academic emotion. The items were assessed on a 5-point scale ranging from 0 (Not at all) to 4 (Very strong; e.g., “To what extent did you experience ___ during the past few weeks of learning?” “I felt…” “angry”).

#### Student Engagement

The ten-item Engagement Questionnaire (Skinner et al., 2009) was used to assess student engagement with respect to students’ behavioral and emotional engagement. For example, “I pay attention in class” was used to assess the behavioral aspect and “Class is fun” assessed the emotional aspect. Each measure employed a 5-point scale ranging from 1 (Not at all true) to 5 (Very true). The scale was internally consistent (α_t__1__/t__2_ = 0.96/0.96).

### Data Analysis

Data were analyzed with SPSS Version 25.0. PROCESS macro for SPSS was adopted to perform the multiple mediation model (Hayes, 2013). Model 82 was used in PROCESS to test the mediating role of positive and negative academic emotions (mediator) in the relationship between adaptability (independent variable) and student engagement (dependent variable). We used 5000 bootstrap samples and the 95% bias-corrected confidence interval (95% CI) to examine the significance of the multiple mediation effect (Hayes, 2013). The statistical significance level was set at *p* < 0.05.

## Results

### Common Method Biases

The Harman single-factor test was used to diagnose the common method bias (Podsakoff et al., 2003). The results of principal component factor analysis without rotation showed that there were 11 factors whose eigenvalues were greater than 1. The variance explained by the first factor was 36.07%, below the threshold of 40%. Therefore, the common method bias did not affect the outcome of this study.

### Descriptive Statistics and Correlations

The means (*M*), standard deviations (*SD*), skewness, and kurtosis, and reliabilities for all variables across the two points are displayed in Table 1. All the measures had acceptable reliabilities (ranging from 0.88 to 0.97).

**TABLE 1:** Descriptive statistics of all study variables (*n* = 1119).

	***M***	***SD***	**Skewness**	**Kurtosis**	**α**
(1) T1 Adaptability	5.22	1.06	–0.65	1.15	0.97
(2) T1 Positive academic emotion	2.80	0.79	0.08	0.70	0.88
(3) T2 Positive academic emotion	2.85	0.85	0.13	0.34	0.90
(4) T1 Negative academic emotion	1.85	0.70	0.96	0.46	0.96
(5) T2 Negative academic emotion	1.87	0.79	1.02	0.65	0.97
(6) T2 Student engagement	3.59	0.80	–0.13	0.02	0.96

*Adaptability (range: 1 to 7), academic emotion (range: 0 to 4), student engagement (range: 1 to 5).*

Pearson correlation matrices for the longitudinal relations between variables are displayed in Table 2. T1 Adaptability was positively correlated with T1 and T2 positive academic emotion (*rs* = 0.29 and 0.19, respectively; *ps* < 0.01) and negatively correlated with T1 and T2 negative academic emotion (*rs* = −0.32 and −0.34, respectively; *ps* < 0.01). T2 student engagement was also positively correlated with T1 and T2 positive academic emotion (*rs* = 0.22 and 0.21, respectively; *ps* < 0.01), but negatively correlated with T1 and T2 negative academic emotion (*rs* = −0.35 and −0.39, respectively; *ps* < 0.01). Furthermore, the correlation between T1 Adaptability and T2 student engagement was significant (*r* = 0.47, *p* < 0.01).

**TABLE 2:** Pearson correlations for all study variables (*n* = 1119).

	**1**	**2**	**3**	**4**	**5**	**6**
(1) T1 Adaptability	–					
(2) T1 Positive academic emotion	0.29**	–				
(3) T2 Positive academic emotion	0.19**	0.45**	–			
(4) T1 Negative academic emotion	−0.32**	−0.13**	−0.10**	–		
(5) T2 Negative academic emotion	−0.34**	−0.13**	−0.07*	0.65**	–	
(6) T2 Student engagement	0.47**	0.22**	0.21**	−0.35**	−0.39**	–

*p < 0.05, **p < 0.01.

### The Multiple Mediation Effects of Adaptability, Academic Emotion, and Student Engagement

**FIGURE 1:**
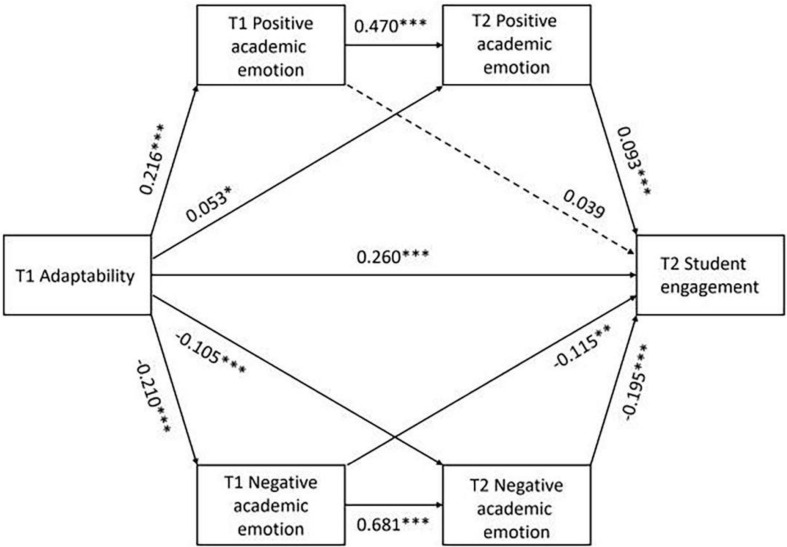
Multiple-mediating test of adaptability, academic emotions, and student engagement.

The results of the regression analyses are shown in Figure 1 and Table 3. T1 adaptability positively predicted T2 student engagement (β = 0.260, *p* < 0.001), as well as T1 positive academic emotion (β = 0.216, *p* < 0.001) and T2 positive academic emotion (β = 0.053, *p* < 0.05). T1 adaptability also negatively predicted T1 negative academic emotion (β = −0.211, *p* < 0.001) and T2 negative academic emotion (β = −0.105, *p* < 0.001). T1 positive academic emotion significantly predicted T2 positive academic emotion (β = 0.470, *p* < 0.001), however, T1 positive academic emotion did not predict T2 student engagement (β = 0.039, *p* = 0.193). A strong regression path was shown between T2 positive academic emotion and T2 student engagement (β = 0.093, *p* < 0.001), between T1 negative academic emotion and T2 negative academic emotion (β = 0.618, *p* < 0.001), and between T1 negative academic emotion and T2 student engagement (β = −0.115, *p* < 0.01).

**TABLE 3:** Regression analysis of variable relationships (*n* = 1119).

**Dependent**	**Predictors**	**Model Summary**				
		***F***	***R*^2^**	**β**	**SE**	***t***
T1 Positive academic emotion	T1 Adaptability	104.97***	0.09	0.216	0.02	10.25
T2 Positive academic emotion	T1 Adaptability	147.84***	0.21	0.053	0.02	2.40
	T1 Positive academic emotion			0.470	0.03	15.58
T1 Negative academic emotion	T1 Adaptability	126.25***	0.10	–0.210	0.02	–11.24
T2 Negative academic emotion	T1 Adaptability	445.77***	0.44	–0.105	0.02	–6.00
	T1 Negative academic emotion			0.681	0.03	25.81
T2 Student engagement	T1 Adaptability	96.00***	0.30	0.260	0.02	12.37
	T1 Positive academic emotion			0.039	0.03	1.30
	T2 Positive academic emotion			0.093	0.03	3.51
	T1 Negative academic emotion			–0.115	0.04	–3.02
	T2 Negative academic emotion			–0.195	0.03	–5.72

***p < 0.001.

In order to test the intermediary role of academic emotion in the relationship between adaptability and student engagement, the Bootstrap method was used to sample 5000 times and build a 95% unbiased correction confidence interval. The results show that the chain intermediary effect of T1 and T2 positive academic emotion (β = 0.010, 95% CI [0.003, 0.017]), and T1 and T2 negative academic emotions (β = 0.028, 95% CI [0.016, 0.042]) were significant, indicating a significant mediation by both positive and negative emotions. The model results are shown in Table 4. In addition, T1 adaptability had indirect effects on T2 engagement through T2 positive academic emotion (β = 0.005, 95% CI [0.000, 0.012]), T1 negative academic emotion (β = 0.024, 95% CI [0.007, 0.043]), and T2 negative academic emotion (β = 0.021, 95% CI [0.011, 0.032]).

**TABLE 4:** Mediation effects test.

	**Ind**	**Indirect effect**			
		**Effect**	**95% confidence interval**		**Percentage**
			**BootLLCI**	**BootULCI**	
Total	Total	0.096	0.073	0.120	26.90%
Ind 1	T1 Adaptability→T1 Positive academic emotion→T2 Student engagement	0.008	–0.006	0.024	2.33%
Ind 2	T1 Adaptability→T2 Positive academic emotion→T2 Student engagement	0.005	0.000	0.012	1.41%
Ind 3	T1 Adaptability→T1 Negative academic emotion→T2 Student engagement	0.024	0.007	0.043	6.82%
Ind 4	T1 Adaptability→T2 Negative academic emotion→T2 Student engagement	0.021	0.011	0.032	5.77%
Ind 5	T1 Adaptability→T1 Positive academic emotion→T2 Positive academic emotion→T2 Student engagement	0.010	0.003	0.017	2.68%
Ind 6	T1 Adaptability→T1 Negative academic emotion→T2 Negative academic emotion→T2 Student engagement	0.028	0.016	0.042	7.89%

*Ind 1 = T1 Adaptability→T1 Positive academic emotion→T2 Student engagement; Ind 2 = T1 Adaptability→T2 Positive academic emotion→T2 Student engagement; Ind 3 = T1 Adaptability→T1 Negative academic emotion→T2 Student engagement; Ind 4 = T1 Adaptability→T2 Negative academic emotion→T2 Student engagement; Ind 5 = T1 Adaptability→T1 Positive academic emotion→T2 Positive academic emotion→T2 Student engagement; Ind 6 = T1 Adaptability→T1 Negative academic emotion→T2 Negative academic emotion→T2 Student engagement.*

## Discussion

In the present study, a multiple mediating model was tested to examine the relations among university students’ adaptability, positive and negative academic emotions, and student engagement during the COVID-19 pandemic in China. The results of this study suggest that adaptability can directly affect student engagement, and it can also indirectly influence student engagement through academic emotion.

Consistent with our expectations concerning the importance of adaptability for student engagement, adaptability was found to influence student engagement positively, supporting the behavioral function of adaptability as a propensity that helps individuals to adjust to the demands in their environment (Martin et al., 2013). This result was in line with a previous study (Susana et al., 2015) that showed that students who can adjust to different situations and circumstances are more likely to engage in learning activities. In the novel and challenging situation for Chinese university students after the outbreak of COVID-19, students who are higher in adaptability were better to engage in sustained efforts to cope with new challenges, keep track of their academic work, and adjust their behaviors to manage new learning tasks. Importantly, the present study suggests this level of adaptability positively predicted students’ levels of engagement, and thus may lead to different levels of academic performance during COVID-19.

Moreover, our results also speak to the emotional function of adaptability, as adaptability predicted student engagement through academic emotion. Successfully adapting in an academic domain can help students feel effective and capable of achieving academic goals, perceive connections with teachers, and therefore produce pleasant emotions and decrease unpleasant emotions in learning (Rahman et al., 2009). We found that students who were best able to adapt to the impact of COVID-19 experienced more positive academic emotions. Positive academic emotions like enjoyment can direct students to engage in academic tasks, which can improve their performance (e.g., Ketonen et al., 2016). Pride is another positive academic emotion that can strengthen students’ long-term motivation to pursue academic goals (Pekrun et al., 2002; Schwarzer and Taubert, 2002). Most importantly, students with positive academic emotions are more willing to invest effort into learning, reaching higher levels of engagement (King et al., 2015). Students’ level of adaptability further predicted their negative emotions, and we found an even stronger impact of adaptability on negative academic emotion than on positive academic emotion. Previous research has identified that negative academic emotions can narrow students’ cognitive scope by making them focus on threats or failure, and in turn pull their limited cognitive resources away from the academic tasks at hand (Owens et al., 2012; Ouweneel et al., 2014).

There are some limitations to this study. Firstly, although the current study is longitudinal, the interval between the two time points was short, at only 2 weeks. Because it was unknown how long the online learning changes due to COVID-19 would last, the second survey was carried out 2 weeks after the first to capture the dynamic development of students’ learning activities during this time. Secondly, the participants were university students in China, so although they came from more than 20 provinces of China, the generalizability to populations in other cultures should be made with caution. Future research should replicate this model in university students in other regions of the world.

Another interesting future direction would be to examine how university students’ health risk perceptions impact their academic emotions and engagement. Both the antecedents (Commodari et al., 2020) and the consequences (Ding et al., 2020) of health risk perceptions during COVID-19 have been examined by recent research. For example, Commodari and La Rosa (2020) examined the relationship between COVID-19 health risk perceptions and emotions among quarantined adolescents in Italy. They found perceived susceptibility negatively predicted positive emotions whereas fear of getting COVID-19 positively predicted negative emotions. The present research suggests that positive and negative emotions resulting from health risk perceptions may impact students’ academic engagement. Future research should further examine the role of students’ adaptability in responding to information regarding risk in addition to adapting to changes in the academic environment.

## Conclusion

The present longitudinal study examined the behavioral and emotional function of adaptability. The results suggest that adaptability under COVID-19 directly increases student engagement (behavioral perspective), and indirectly promotes student engagement by enhancing positive academic emotion and dampening negative academic emotions (emotional perspective). This study provides an important preliminary understanding of how university students’ adaptability influences their academic engagement via academic emotions under COVID-19. Thus, the present findings have relevance to efforts to understand how to support students to successfully navigate challenging environmental conditions and promote effective adjustment to challenges, including global pandemics.

## Data Availability Statement

The datasets presented in this study can be found in online repositories. The names of the repository/repositories and accession number(s) can be found below: https://osf.io/3k7v9/?view_only=e5738638d1244f66bd3df8548ee46452.

## Ethics Statement

The studies involving human participants were reviewed and approved by the Qingdao University. Written informed consent to participate in this study was provided by the participants’ legal guardian/next of kin.

## Author Contributions

KZ and SW conceived and designed the survey, performed the survey, and contributed materials and analysis tools. KZ and YX analyzed the data. KZ, SW, YX, WC, TG, and EP-S wrote the manuscript. KZ, YX, and EP-S literature research. All authors contributed to the article and approved the submitted version.

## Conflict of Interest

The authors declare that the research was conducted in the absence of any commercial or financial relationships that could be construed as a potential conflict of interest.

## Footnotes

1 http://www.moe.gov.cn/

2 https://www.wjx.cn/

## References

[B1] Appleton J. J. Christenson S. L. Furlong M. J. (2008). Student engagement with school: critical conceptual and methodological issues of the construct. *Psychol. Sch.* 45 369–386. 10.1002/pits.20303

[B2] Besser A. Flett G. L. Zeigler-Hill V. (2020). Adaptability to a sudden transition to online learning during the COVID-19 pandemic: understanding the challenges for students. *Scholarsh. Teach. Learn. Psychol.* 10.1037/stl0000198 [Epub ahead of print].

[B3] Boulton C. A. Hughes E. Kent C. Smith J. R. Williams H. T. P. (2019). Student engagement and wellbeing over time at a higher education institution. *PLoS One* 14:e0225770. 10.1371/journal.pone.0225770 31774878 PMC6881016

[B4] Cao W. Fang Z. Hou G. Han M. Xu X. Dong J. (2020). The psychological impact of the COVID-19 epidemic on college students in China. *Psychiatry Res.* 287:112934. 10.1016/j.psychres.2020.112934 32229390 PMC7102633

[B5] Chen R. N. Liang S. W. Peng Y. Li X. G. Zhao J. B. (2020). Mental health status and change in living rhythms among college students in China during the COVID-19 pandemic: a large-scale survey. *J. Psychosom. Res.* 137:110219. 10.1016/j.jpsychores.2020.110219 PMC7428432 32862063

[B6] Collie R. J. Holliman A. J. Martin A. J. (2016). Adaptability, engagement and academic achievement at university. *Educ. Psychol.* 37 632–647. 10.1080/01443410.2016.1231296

[B7] Collie R. J. Martin A. J. (2017). Students’ adaptability in mathematics: examining self-reports and teachers’ reports and links with engagement and achievement outcomes. *Contemp. Educ. Psychol.* 49 355–366. 10.1016/j.cedpsych.2017.04.001

[B8] Commodari E. La Rosa V. L. (2020). Adolescents in quarantine during COVID-19 pandemic in Italy: perceived health risk, beliefs, psychological experiences and expectations for the future. *Front. Psychol.* 11:2480. 10.3389/fpsyg.2020.559951 33071884 PMC7538632

[B9] Commodari E. La Rosa V. L. Coniglio M. A. (2020). Health risk perceptions in the era of the new coronavirus: are the Italian people ready for a novel virus? A cross-sectional study on perceived personal and comparative susceptibility for infectious diseases. *Public Health* 187 8–14. 10.1016/j.puhe.2020.07.036 32866818 PMC7396885

[B10] Derakshan N. Smyth S. Eysenck M. W. (2009). Effects of state anxiety on performance using a task-switching paradigm: an investigation of attentional control theory. *Psychon. Bull. Rev.* 16 1112–1117. 10.3758/PBR.16.6.1112 19966264

[B11] Ding Y. Xu J. Huang S. Li P. Lu C. Xie S. (2020). Risk perception and depression in public health crises: evidence from the COVID-19 crisis in China. *Int. J. Environ. Res. Public Health* 17:5728. 10.3390/ijerph17165728 32784792 PMC7460398

[B12] Farooq F. Rathore F. A. Mansoor S. N. (2020). Challenges of online medical education in Pakistan during COVID-19 pandemic. *J. Coll. Physicians Surg. Pak.* 30, 67–69. 10.29271/jcpsp.2020.Supp1.S67 32723456

[B13] Fredricks J. A. McColskey W. (2012). “The measurement of student engagement: a comparative analysis of various methods and student self-report instruments,” in *Handbook of Research on Student Engagement*, eds Christenson S. L. Reschly A. L. Wylie C. (Boston, MA: Springer US), 763–782. 10.1007/978-1-4614-2018-7_37

[B14] Fredrickson B. L. (2001). The role of positive emotions in positive psychology. The broaden-and-build theory of positive emotions. *Am. Psychol.* 56 218–226. 10.1037//0003-066x.56.3.218 11315248 PMC3122271

[B15] Fredrickson B. L. Joiner T. (2018). Reflections on positive emotions and upward spirals. *Perspect. Psychol. Sci.* 13 194–199. 10.1177/1745691617692106 29592643 PMC5877808

[B16] Goetz T. Frenzel A. C. Hall N. C. Pekrun R. (2008). Antecedents of academic emotions: testing the internal/external frame of reference model for academic enjoyment. *Contemp. Educ. Psychol.* 33 9–33. 10.1016/j.cedpsych.2006.12.002

[B17] Guan W. Ni Z. Hu Y. Liang W. Ou C. He J. (2020). Clinical characteristics of coronavirus disease 2019 in China. *N. Engl. J. Med.* 382 1708–1720. 10.1056/NEJMoa2002032 32109013 PMC7092819

[B18] Hayes A. (2013). Introduction to mediation, moderation, and conditional process analysis. *J. Educ. Meas.* 51 335–337. 10.1111/jedm.12050

[B19] Holliman A. J. (2013). *The Routledge International Companion to Educational Psychology.* Abingdon: Routledge.

[B20] Holliman A. J. Martin A. J. Collie R. J. (2018). Adaptability, engagement, and degree completion: a longitudinal investigation of university students. *Educ. Psychol.* 38 785–799. 10.1080/01443410.2018.1426835

[B21] Izard C. E. Dougherty F. E. Bloxom B. M. Kotsch W. E. (1974). *The Differential Emotions Scale: A Method of Measuring the Subjective Experience of Discrete Emotions.* Nashville, TN: Vanderbilt University.

[B22] Jiang H.-J. Nan J. Lv Z.-Y. Yang J. (2020). Psychological impacts of the COVID-19 epidemic on Chinese people: exposure, post-traumatic stress symptom, and emotion regulation. *Asian Pac. J. Trop. Med.* 13 252–259.

[B23] Ketonen E. E. Haarala-Muhonen A. Hirsto L. Hänninen J. J. Wähälä K. Lonka K. (2016). Am I in the right place? Academic engagement and study success during the first years at university. *Learn. Individ. Dif.* 51 141–148. 10.1016/j.lindif.2016.08.017

[B24] King R. B. Gaerlan M. J. M. (2014). How you Perceive Time Matters for how you Feel in School: investigating the link between time perspectives and academic emotions. *Curr. Psychol.* 33 282–300. 10.1007/s12144-014-9213-x

[B25] King R. B. Mcinerney D. M. Ganotice F. A. Villarosa J. B. (2015). Positive affect catalyzes academic engagement: cross-sectional, longitudinal, and experimental evidence. *Learn. Individ. Dif.* 39 64–72. 10.1016/j.lindif.2015.03.005

[B26] Martin A. J. Nejad H. Colmar S. Liem G. A. D. (2012). Adaptability: conceptual and empirical perspectives on responses to change, Novelty and Uncertainty. *Aust. J. Guid. Coun.* 22 58–81. 10.1017/jgc.2012.8

[B27] Martin A. J. Nejad H. G. Colmar S. Liem G. A. D. (2013). Adaptability: how students’ responses to uncertainty and novelty predict their academic and non-academic outcomes. *J. Educ. Psychol.* 105 728–746. 10.1037/a0032794

[B28] Nickerson L. A. Shea K. M. (2020). First-Semester organic chemistry during COVID-19: prioritizing group work, flexibility, and student engagement. *J. Chem. Educ.* 97 3201–3205. 10.1021/acs.jchemed.0c00674

[B29] Ouweneel E. Le Blanc P. M. Schaufeli W. B. (2014). On being grateful and kind: results of two randomized controlled trials on study-related emotions and academic engagement. *J. Psychol.* 148 37–60. 10.1080/00223980.2012.742854 24617270

[B30] Owens M. Stevenson J. Hadwin J. A. Norgate R. (2012). Anxiety and depression in academic performance: an exploration of the mediating factors of worry and working memory. *Sch. Psychol. Int.* 33 433–449. 10.1777/0143034311427433

[B31] Pekrun R. Goetz T. Titz W. Perry R. P. (2002). Academic emotions in students’ self-regulated learning and achievement: a program of qualitative and quantitative research. *Educ. Psychol.* 37 91–105. 10.1207/S15326985EP3702_4

[B32] Perets E. A. Chabeda D. Gong A. Z. Huang X. Fung T. S. Ng K. Y. (2020). Impact of the emergency transition to remote teaching on student engagement in a Non-STEM undergraduate chemistry course in the time of COVID-19. *J. Chem. Educ.* 97 2439–2447. 10.1021/acs.jchemed.0c00879

[B33] Podsakoff P. M. MacKenzie S. B. Lee J.-Y. Podsakoff N. P. (2003). Common method biases in behavioral research: a critical review of the literature and recommended remedies. *J. Appl. Psychol.* 88 879–903. 10.1037/0021-9010.88.5.879 14516251

[B34] Putwain D. Sander P. Larkin D. (2013). Academic self-efficacy in study-related skills and behaviours: relations with learning-related emotions and academic success. *Br. J. Educ. Psychol.* 83 633–650. 10.1111/j.2044-8279.2012.02084.x 24175686

[B35] Rahman R. J. Thøgersenntoumani C. Thatcher J. Doust J. (2009). Psychological need satisfactions as predictors of motivation and psychological outcomes following exercise rehabilitation. *Psychol. Health* 24 330–330.

[B36] Reschly A. Huebner E. Appleton J. Antaramian S. (2008). Engagement as flourishing: the contribution of positive emotions and coping to adolescents’ engagement at school and with learning. *Psychol. Sch.* 45 419–431. 10.1002/pits.20306

[B37] Restubog S. L. D. Ocampo A. C. G. Wang L. (2020). Taking control amidst the chaos: emotion regulation during the COVID-19 pandemic. *J. Vocat. Behav.* 119:103440 10.1016/j.jvb.2020.103440 PMC7206430 32390659

[B38] Schwarzer R. Taubert S. (2002). “Tenacious goal pursuits and striving toward personal growth: proactive coping,” in *Beyond Coping: Meeting Goals, Visions and Challenges*, ed. Frydenberg E. (Oxford: Oxford University Press), 19–35. 10.1093/med:psych/9780198508144.003.0002

[B39] Skinner E. A. Kindermann T. A. Furrer C. J. (2009). A motivational perspective on engagement and disaffection conceptualization and assessment of children’s behavioral and emotional participation in academic activities in the classroom. *Educ. Psychol. Meas.* 69 493–525. 10.1177/0013164408323233

[B40] Steele J. P. Fullagar C. J. (2009). Facilitators and outcomes of student engagement in a college setting. *J. Psychol.* 143 5–27. 10.3200/JRLP.143.1.5-27 19157070

[B41] Susana C. Mats G. Lena A. (2015). The relationship between classroom quality-related variables and engagement levels in Swedish preschool classrooms: a longitudinal study. *Eur. Early Child. Educ. Res. J.* 25 122–135. 10.1080/1350293x.2015.1102413

[B42] VandenBos G. R. (ed.) (2007). *). American Psychological Association Dictionary of Psychology.* Washington, DC: American Psychological Association.

[B43] Wang C. Pan R. Wan X. Tan Y. Xu L. McIntyre R. S. (2020). A longitudinal study on the mental health of general population during the COVID-19 epidemic in China. *Brain Behav. Immun.* 87 40–48. 10.1016/j.bbi.2020.04.028 32298802 PMC7153528

[B44] Wang C. Zhao H. (2020). The impact of COVID-19 on anxiety in Chinese university students. *Front. Psychol.* 11:1168. 10.3389/fpsyg.2020.01168 32574244 PMC7259378

[B45] Wu Z. McGoogan J. M. (2020). Characteristics of and important lessons from the coronavirus disease 2019 (COVID-19) Outbreak in China summary of a report of 72 314 cases from the Chinese center for disease control and prevention. *Jama J. Am. Med. Assoc.* 323 1239–1242. 10.1001/jama.2020.2648 32091533

[B46] Zhen R. Liu R.-D. Ding Y. Wang J. Liu Y. Xu L. (2017). The mediating roles of academic self-efficacy and academic emotions in the relation between basic psychological needs satisfaction and learning engagement among Chinese adolescent students. *Learn. Indiv. Dif.* 54 210–216. 10.1016/j.lindif.2017.01.017

[B47] Zhou F. Yu T. Du R. Fan G. Liu Y. Liu Z. (2020). Clinical course and risk factors for mortality of adult inpatients with COVID-19 in Wuhan, China: a retrospective cohort study. *Lancet* 395 1054–1062. 10.1016/s0140-6736(20)30566-3 32171076 PMC7270627

